# Ultramicroscopy as a novel tool to unravel the tropism of AAV gene therapy vectors in the brain

**DOI:** 10.1038/srep28272

**Published:** 2016-06-20

**Authors:** Sandro Alves, Julia Bode, Alexis-Pierre Bemelmans, Christof von Kalle, Nathalie Cartier, Björn Tews

**Affiliations:** 1 INSERM U1169/MIRCen CEA, Fontenay aux Roses 92265, France, Université Paris-Sud, Université Paris-Saclay, Orsay 91400, France; 2 Schaller Research Group at the University of Heidelberg and the German Cancer Research Center (DKFZ), Im Neuenheimer Feld 581, 69120 Heidelberg, Germany; 3 Molecular Mechanisms of Tumor Invasion (V077), DKFZ, Im Neuenheimer Feld 581, 69120 Heidelberg, Germany; 4 Commissariat à l´Energie Atomique et aux Energies Alternatives (CEA), Départment de la Recherche Fondamentale (DRF), Institut d´Imagerie Biomédicale (I2BM), Molecular Imaging Research Center (MIRCen), Fontenay-aux-Roses, France; 5 Centre National de la Recherche Scientifique (CNRS), Université Paris-Sud, Université Paris-Saclay, UMR 9199, Neurodegenerative Diseases Laboratory, Fontenay-aux Roses, France; 6 Department of Translational Oncology, National Center for Tumor Diseases (NCT) and German Cancer Research Center (DKFZ), Im Neuenheimer Feld 280, 69120 Heidelberg, Germany

## Abstract

Recombinant adeno-associated viral (AAV) vectors have advanced to the vanguard of gene therapy. Numerous naturally occurring serotypes have been used to target cells in various tissues. There is a strong need for fast and dynamic methods which efficiently unravel viral tropism in whole organs. Ultramicroscopy (UM) is a novel fluorescence microscopy technique that images optically cleared undissected specimens, achieving good resolutions at high penetration depths while being non-destructive. UM was applied to obtain high-resolution 3D analysis of AAV transduction in adult mouse brains, especially in the hippocampus, a region of interest for Alzheimer’s disease therapy. We separately or simultaneously compared transduction efficacies for commonly used serotypes (AAV9 and AAVrh10) using fluorescent reporter expression. We provide a detailed comparative and quantitative analysis of the transduction profiles. UM allowed a rapid analysis of marker fluorescence expression in neurons with intact projections deep inside the brain, in defined anatomical structures. Major hippocampal neuronal transduction was observed with both vectors, with slightly better efficacy for AAV9 in UM. Glial response and synaptic marker expression did not change post transduction.We propose UM as a novel valuable complementary tool to efficiently and simultaneously unravel tropism of different viruses in a single non-dissected adult rodent brain.

Viral vectors enable a controlled spatiotemporal expression of transgenes of interest in several tissues and have become a widely used vehicle for gene transfer in biological sciences, including neurobiology^1^. In the last few years, recombinant viral vectors derived from adeno-associated virus (AAV) have evolved as an important and reliable tool for gene transfer^2^. AAVs are very small non-enveloped single-stranded DNA viruses with a tiny capsid (~22 nm). AAVs belong to the family of *Parvoviridae* and the genus *Dependovirus* due to their incompetence to complete their life cycle and replicate in the absence of other helper viruses^3^. AAVs were first described in 1965 as a concomitant of adenoviral stocks^4^. The suitability of AAVs as a mammalian vehicle for gene transfer was first demonstrated by Hermonat and co-workers^5^. Since then, the wild-type AAV capsid coding region suffered successive alterations generating robust recombinant AAVs that were demonstrated to efficiently transduce mammalian cells^6^. Several preclinical and clinical studies have been carried out with the first approved human gene therapy product, Glybera, an AAV1-based gene therapy that has been developed for the treatment of patients with lipoprotein lipase (LPL) deficiency. AAVs also serve as preferred vectors in current clinical trials for gene therapies addressing neurodegenerative diseases, such as Parkinson’s and Alzheimer’s disease (AD)^7^. AAVs are able to transduce dividing as well as non-dividing cells. These viruses enable long-term expression of genes of interest in transduced cells^8^. Over the last ten years, a wide range of naturally occurring AAV serotypes, which mainly vary in the characteristics of the capsid surface, has been identified^9^,^10^. Presently, vector-packaging systems comprising approximately ten different natural serotypes are accessible for the generation of AAV gene therapy vectors^11^,^12^, depending on specific interactions of the capsid proteins within transduced cells. This has been reported in several studies and showed dissimilarities in the transduction efficiency of particular AAV serotypes in different cell types and tissues^13^. Thus, it is crucial to choose the most appropriate serotype to maximize the expression of a certain transgene for a specific application. Therefore, it is mandatory to obtain quantitative results on the transduction properties of different serotypes. Here, we qualitatively and quantitatively compare two different serotypes recently used for gene delivery, namely AAV9 and AAVrh10, combining conventional techniques with Ultramicroscopy (UM)^14^,^15^. UM is a novel fluorescence microscopy technique that applies a focused light sheet to illuminate an optically cleared specimen from the side, e.g., a whole rodent brain, perpendicular to the objective. This technique achieves excellent resolution at high penetration depths while being non-destructive at the same time. Further advantages are strongly reduced photo-bleaching effects, high dynamic range, and rapid acquisition speeds. UM enabled detailed analysis of AAV9- and AAVrh10-mediated GFP or tdTomato reporter gene expression in whole adult mouse brains on a single cell level. We specifically addressed cellular tropism of the pseudotyped AAVs in the hippocampus, a prime region of interest as a therapeutic target for gene therapy in AD. We also assessed the potential of the AAV vectors to induce activation of glial responses and expression of synaptic markers by classical immunohistochemistry and western blot. We propose UM as a valuable complementary tool to efficiently unravel viral tropism in non-dissected intact whole organs.

## Results

### Widespread GFP expression following AAV9 and AAVrh10 transduction of the adult mouse hippocampus

**Figure 1:**
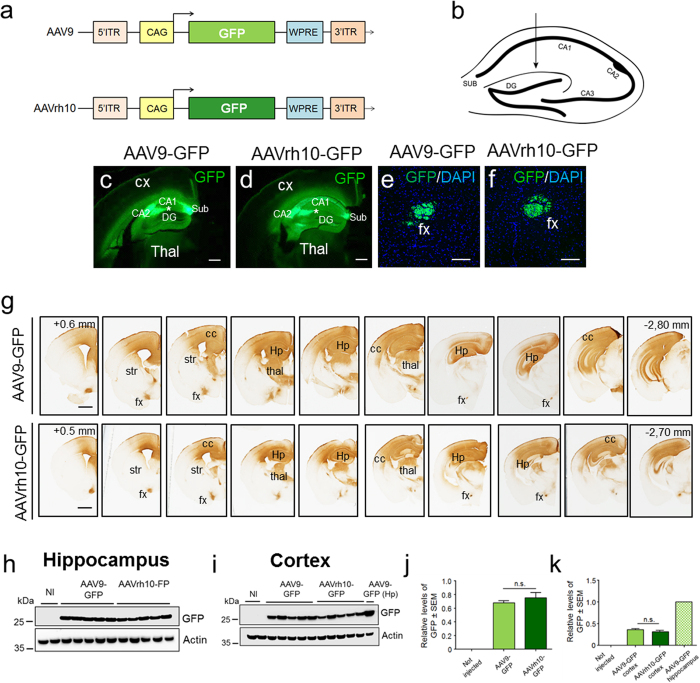
AAV9 and AAVrh10 allow widespread GFP distribution in the adult mouse hippocampus. (**a**) Schematic representation of AAV9 and AAVrh10 constructs enabling the expression of the green fluorescent protein (GFP) driven by the hybrid cytomegalovirus enhancer/chicken beta-actin constitutive promoter (CAG). ITR: inverted terminal repeat; WPRE: Woodchuck hepatitis virus posttranscriptional regulatory element. Two-month old C57BL/6J mice were stereotactically injected with AAV9-GFP or AAVrh10-GFP in the mouse hippocampus (**b**). GFP fluorescence in hippocampal slices showing similar pattern of GFP fluorescence and layer distribution among AAV9-GFP (**c**) and AAVrh10-GFP (**d**) injected mice. Immunoreactivity was stronger in the CA2 cell layer compared to the CA1 layer. Similar levels of GFP were also detected in the subiculum (Sub) and dentate gyrus (**c**,**d**) in the hippocampi transduced with AAV9-GFP or AAVrh10-GFP vectors. Laser confocal microscopy showing GFP-positive fibres projecting from the site of injection within the hippocampal commissure and dorsal fornix (fx) in the mouse hippocampi transduced with AAV9 (**e**) or AAVrh10 (**f**,**g**) Immunohistochemical labelling of the GFP protein showing equivalent and widespread transduction of the GFP protein, in hippocampal (Hp) and cortical regions (cc) from mice receiving AAV9-GFP or AAVrh10-GFP (Str, striatum; Thal, thalamus). (**h**) Western blot of hippocampal lysates of mice injected with AAV9-GFP or AAVrh10-GFP showing similar levels of GFP. Non-injected mice were used as control. Actin was used as a loading control. (**i**) Western blot of lysates from the cerebral cortex showing similar levels of GFP when overexpressed using AAV9 or AAVrh10 vectors. (**j,k**) Optical densitometries representative of western blot of hippocampal (**h**) or the respective cortical biopsies (**i**). Optical densitometry was normalized according to the amount of actin loaded in the corresponding lane. A partition ratio was calculated and expressed as optical densitometry (arbitrary units) relative to the sample with highest value for the normalization control set at 1. Values are expressed as mean ± SEM. Student’s t-test; representative data from 5 mice/group. Bars: (**d**,**e**)- 500 μm; (**f**,**g**) 100 μm, (**h**) 1 mm.

AAV9 and AAVrh10 vector stocks encoding the GFP reporter under control of the chicken β-actin/cytomegalovirus (CMV) hybrid (CAG) promoter (Fig. 1a), that is known for its high neuron-specificity after direct AAV injections to the adult CNS^16^, were titre-matched. A volume of 2 μl containing a total of 2 × 10^9^ vg was injected bilaterally into the adult mouse hippocampus (Fig. 1b). Hippocampal and cortical biopsies were harvested six weeks post-injection and examined either for the presence of visible GFP fluorescence or were used for GFP-directed immunohistochemistry and western blot analysis, respectively (Fig. 1c–l).

In order to study the pattern of GFP biodistribution in the mouse hippocampus, we analysed direct GFP fluorescence in brain slices from mice injected with AAV9 or AAVrh10 encoding the GFP protein (Fig. 1c–f). In general, the pattern of GFP fluorescence was similar among AAV9-GFP (n = 5) (Fig. 1c) and AAVrh10-GFP (n = 5) (Fig. 1d) injected hippocampi. In addition, we found a differential pattern of GFP immunoreactivity in different sub-regions of the hippocampus; in both cases immunoreactivity was stronger in the stretch of fibres projecting from the *strata lacunosa moleculare* (site of injection; see asterisk) along the CA2 cell layer, relative to the CA1 layer, where GFP immunoreactivity was present, however with lower intensity (Fig. 1c,d). The hippocampal subiculum, as well as the dentate gyrus from mouse hippocampi transduced with AAV9-GFP or AAVrh10-GFP vectors, revealed high levels of GFP direct fluorescence with no significant differences between these two AAV vectors (Fig. 1c,d). Furthermore, by using confocal microscopy, we found GFP-positive fibres projecting from the site of injection within the hippocampal commissure and dorsal fornix in mice in which the hippocampus was transduced with either AAV9 or AAVrh10 (Fig. 1e,f).

Next, we analysed by immunohistochemistry the rostro-caudal distribution of GFP within the hippocampus and neighbouring brain regions. When either AAV9-GFP or AAVrh10-GFP were used to transduce the mouse hippocampus, GFP could be robustly detected from the anterior to the posterior hippocampal structure as well as in several layers of the cerebral cortex (cc), in the thalamus (thal) and in the fornix (fx). Overall, both AAV9 (n = 5) and AAVrh10 (n = 5) allowed a similar and widespread transduction of the GFP protein, which was visible over a region comprising at least ~3.5 mm (antero-posterior) when detected with an anti-GFP antibody (Fig. 1g). GFP immunoreactivity was only faintly detectable in the striatum (str) when AAV9-GFP or AAVrh10-GFP was injected in the mouse hippocampus, however, it could be strongly detected in cortical regions.

We next performed western blot analysis in hippocampal biopsies from mice injected with AAV9-GFP (n = 5) or AAVrh10-GFP (n = 5) in the hippocampus, as well as in non-injected mice. As expected, the transgene was not detectable in non-injected mice; however mice injected with AAV9-GFP and AAVrh10-GFP showed strong and similar levels of GFP expression (Fig. 1h,j). The analysis of biopsies from the cerebral cortex from the same mice also revealed similar expression of the transgene, when the hippocampus was targeted with either AAV9-GFP or AAVrh10-GFP, respectively (Fig. 1I,k). As expected, GFP levels were lower in the cerebral cortex compared to levels obtained in the hippocampi for both AAVs encoding GFP when injected locally (Fig. 1I,k). Altogether, these results indicate that both AAV9 and AAVrh10 allow high and sustained expression of transgenes in several brain regions over large areas.

### UM allows single cell detection of GFP expression in sub-anatomical regions of whole undissected mouse brains

**Figure 2:**
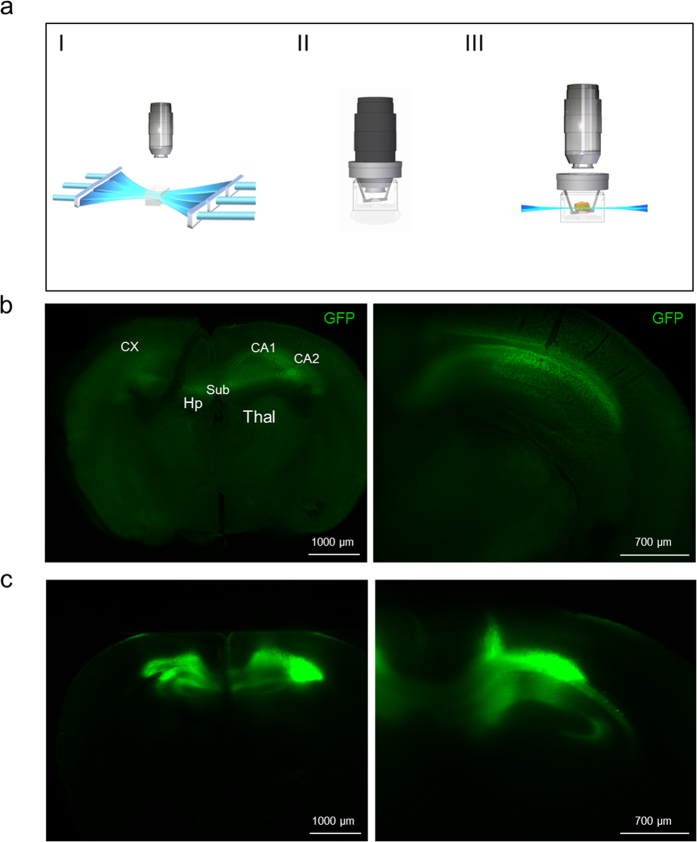
Ultramicroscopy (UM) can detect AAV9 and AAVrh10 distribution in the adult mouse hippocampus. (**a**) Schematic description of the used UM-setup. Bidirectional lasers excite the sample. Different filter settings can be used for the excitation of the sample (I). The objective enters the cuvette and detects the emitted fluorescence perpendicular to the sample (II). The sample is placed in a sample holder in the cuvette and is simultaneously excited with light sheets from the left and right. The objective detects the fluorescence emitted from the sample (III). (**b**) Example of GFP fluorescence in the hippocampal region. A similar GFP pattern can be observed between the CA2- and CA1 cell layer in a 3DISCO cleared AAV9-GFP transduced whole mouse brain. Similar GFP levels were detected in the subiculum and dentate gyrus of hippocampi transduced with AAV9-GFP. Hippocampi of brains transduced with AAVrh10-GFP show similar fluorescence distribution patterns, but with weaker intensities. The brain shown was magnified 2-fold (left) and 4-fold (right) (**c**). Example of an AAV9-GFP transduced brain. FluoClearBABB-cleared whole brains revealed GFP expression in hippocampal regions similar to the 3DISCO-cleared brains. Fluorescence signals were stronger and less background was detectable. GFP fluorescence from the CA1- and CA2 cell layer as well as from the subiculum was detectable in AAV9-GFP and AAVrh10-GFP transduced brains. AAVrh10-GFP transduced brains show less fluorescence signals but hippocampal regions are clearly visible. The brain shown was magnified 2-fold and 4-fold (left; right). Figure 2a I,II and III were kindly provided by LaVision BioTec GmbH, Bielefeld, Germany.

We adapted recently published light sheet microscopy protocols of cleared whole brain specimens^15^,^17^. The endogenous fluorescence of AAV-GFP transduced regions was directly assessed after clearing using an Ultramicroscope as depicted in Fig. 2a. The schematic description of the used UM-setup shows the bidirectional laser excitation of the sample. Different filter settings can be used for the excitation of the sample. The objective enters the cuvette and detects the emitted fluorescence perpendicular to the sample. Strong GFP fluorescence signals in both AAV9- and AAVrh10-transduced brains were clearly visible on a single cell level. UM analysis of cleared whole undissected adult mouse brains, transduced with AAV9-GFP or AAVrh10-GFP, respectively, confirmed the GFP expression pattern obtained with confocal microscopy. UM revealed high transduction of both CA pyramidal neurons and dentate granule neurons with AAV9 and AAVrh10 vectors (Fig. 2b). A comparison of AAV9-GFP and AAVrh10-GFP transduced adult mouse hippocampi revealed that AAV9 is the more efficient shuttle among the investigated serotypes. Fluorescence intensities of 3DISCO and FluoClearBABB cleared whole brains were determined in counts using the same magnification (1.26x) and laser intensity. Fluorescence signals in AAV9-GFP transduced mouse brains were higher in comparison to those obtained from AAVrh10-GFP transduced mouse brains, regardless of which clearing protocol was used (data not shown). 3DISCO-cleared brains transduced with AAV9 showed GFP expression in anterior to posterior hippocampal structures, in several layers of the cerebral cortex and in the thalamus (Fig. 2b). A movie of the whole z-stack is provided in the Supplementary Video S1 showing several layers of the hippocampus transduced with AAV9-GFP. Similar results for anatomical localization of GFP expression were obtained for AAV9 transduced whole brains cleared with the recently published FluoClearBABB protocol, which is especially suited for aged brains (Fig. 2c). When comparing the rapid 3DISCO with the more time-consuming FluoClearBABB protocol (3DISCO: five days *vs*. FluoClearBABB: nine days/whole brain), it is obvious that FluoClearBABB-cleared brains have less background and stronger GFP signals. Thus, GFP-transduced anatomical substructures can be identified more easily (Fig. 2c). The maximum resolution that can be achieved using the UM at hand is 6.3 μm, which allows to identify single cells but not subcellular structures.

The neuronal tropism in the hippocampus is clearly visible in cleared brains (Fig. 2b). A direct comparison of different viral vectors was achieved by transducing different fluorophores with different viral vectors. Co-transduced cells express several reporter fluorescent signals, whereas cells transduced by solely one viral vector express only one particular fluorescent reporter. 2D overlays of these fluorescent signals from multiple channels were generated.

### AAV9 and AAVrh10 display neuronal tropism in the mouse hippocampus and expression of synaptic markers is maintained post transduction

**Figure 3:**
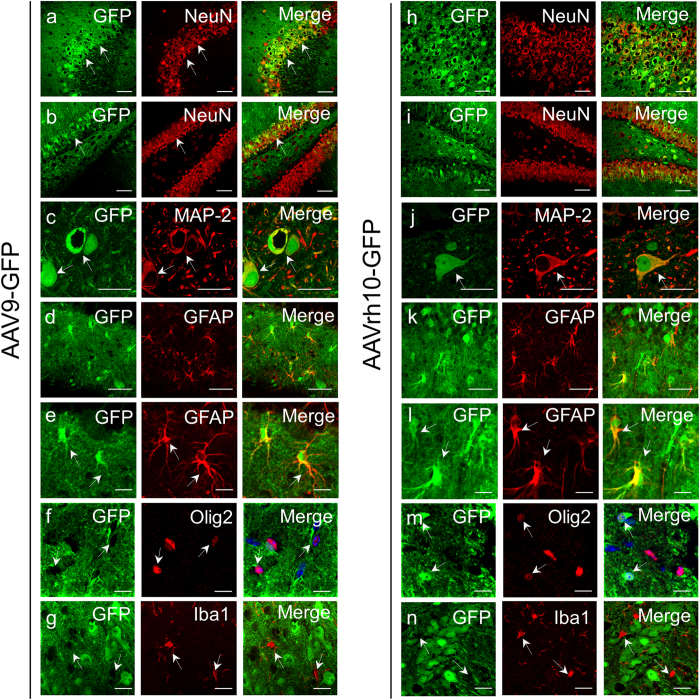
AAV9 and AAVrh10 allow transduction of several cell types, exhibiting preferential neuronal tropism in the mouse hippocampus. Laser confocal microscopy showing predominant co-localization between GFP (in green) and neurons (NeuN, in red) in the hippocampal CA2 layer (**a**) or in the dentate gyrus (**b**) of mice transduced with AAV9-GFP. Similar data were found in mice transduced with AAVrh10-GFP: High neuronal transduction in the CA2 layer or dentate gyrus (**h,i**, respectively). Strong co-localization between GFP (in green) and the neuronal marker MAP-2 (in red) and hippocampal neurons in AAV9-GFP (**c**) or AAVrh10-GFP transduced hippocampi (**j**). Similar astrocytic tropism was also showed within GFP labelling in the hippocampi of mice transduced with AAV9-GFP (**d,e**, high magnification) and AAVrh10-GFP (**k,l**, high magnification). Co-labelling between GFP and Olig2 (in red) revealed poor oligodendrocytes transduction with AAV9-GFP (**f**) and some co-localization between GFP and oligondendrocytes with AAVrh10-GFP (**m**). Double staining between Iba1 and GFP revealed no GFP co-localization within microglial cells with either AAV9-GFP (**g**) or AAVrh10 vectors (**n**). Bars: (**a**,**b**,**h**,**i**) 100 μm; (**c**,**j**) 20 μm; (**d**,**k**) 50 μm, (**e**–**g**,**l**–**n**) 20 μm.

To further characterize the cellular tropism of both AAV9 and AAVrh10 serotypes, we performed double immunostainings of the GFP protein and several markers that specifically label different CNS cell types, i.e., neurons (NeuN and MAP-2), astrocytes (GFAP), microglia (Iba1) and oligodendrocytes (Olig2) in brain slices from mice injected with AAV9-GFP or AAVrh10-GFP. Double immunostained sections were then analysed by confocal microscopy which revealed that most GFP-expressing cells (>80%) were neurons, as demonstrated by the strong co-localization between transgenic GFP protein and NeuN in AAV9-GFP injected mice (Fig. 3a,b) or in AAVrh10-GFP injected mice (Fig. 3h,i). These results were further confirmed by the co-labelling of MAP-2, a neuron-specific cytoskeletal protein, and GFP, where both proteins displayed a strong co-localization in either AAV9-GFP (Fig. 3c) or AAVrh10-GFP settings (Fig. 3j).

We then co-labelled sections that were GFP-positive with antibodies against cell-specific astrocyte (GFAP) and oligodendrocyte (Olig2) markers. The co-immunostaining of GFAP and GFP further revealed the presence of GFP-positive signals in astrocytes in the hippocampus injected with AAV9-GFP (Fig. 3d,e) and AAVrh10 (Fig. 3k,l), respectively. Additionally, we performed co-labelling of GFP and Olig2, which revealed poor transduction of oligodendrocytes with AAV9-GFP (Fig. 3f) and or AAVrh10-GFP (Fig. 3m). Double staining of the microglial marker Iba1 revealed no co-localization with GFP when using AAV9 or AAVrh10 (Fig. 3g,n). These data indicate that both AAV9 and AAVrh10 preferentially exhibit a neuronal tropism; however, non-neuronal cells, such as astrocytes and few oligodendrocytes can also be targeted.

**Figure 4:**
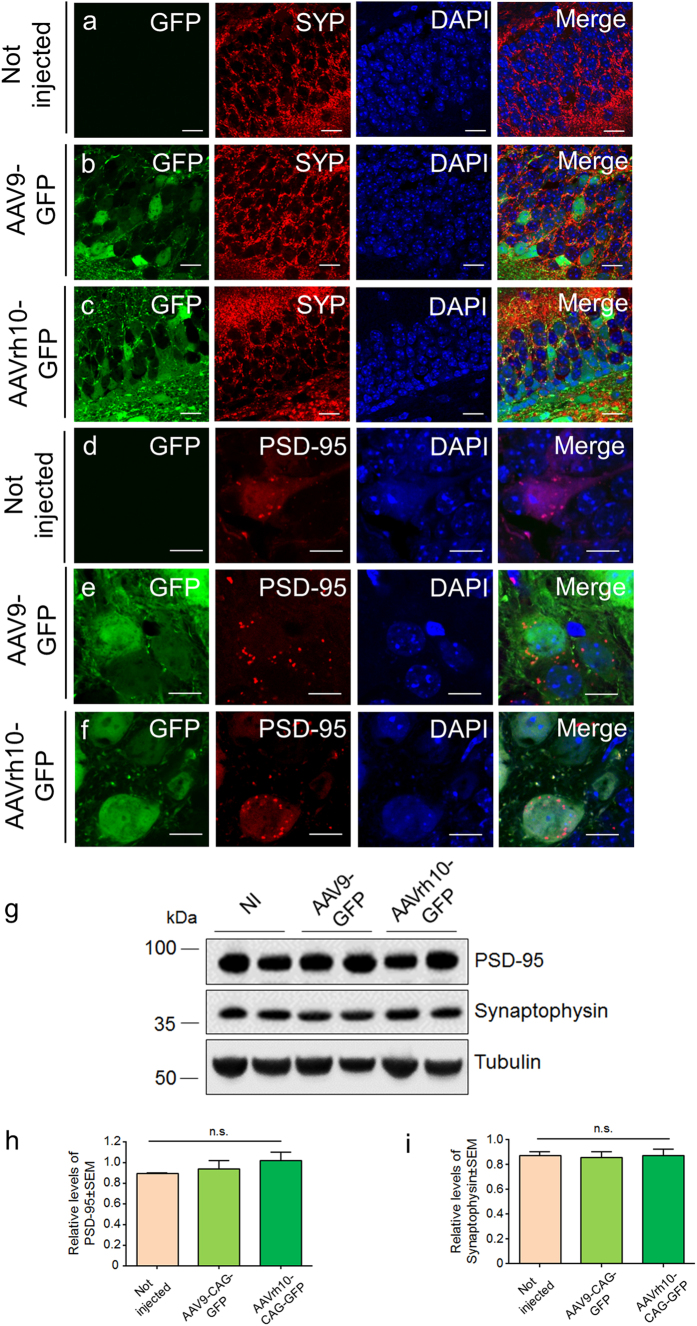
AAV9 and AAVrh10 vectors mediate high hippocampal GFP expression accompanied with preservation of synaptic markers. Laser confocal microscopy representative of double immunostaining between GFP (in green) and synaptophysin (SYP, in red) revealed no major differences in SYP immunoreactivity in non-transduced, AAV9-GFP- or AAVrh10-GFP-transduced hippocampi (**a–c**, respectively), showing abundant presynaptic vesicles (in red). Double immunostaining between PSD-95 (in red) and GFP (in green) showing many PSD-95 positive vesicles did not show major differences between non-transduced, AAV9-GFP- or AAVrh10-GFP-transduced hippocampi (**d–f**, respectively). Bars: (**a**–**c**) 20 μm; (**d**–**f**) 15 μm. Representative western blot of hippocampal lysates injected with AAV9-GFP- or AAVrh10-GFP revealed no statistically significant differences in the levels of PSD-95 and SYP between non-injected mice or mice transduced with AAV9-GFP or AAVrh10-GFP (**g–i**). Student’s t-test. Representative data from 5 mice/group.

Next, we analysed the impact of GFP overexpression on the expression of synaptic markers in hippocampi transduced with AAV9-GFP or AAVrh10-GFP, respectively. For this purpose, we carried out immunohistochemical stainings for synaptophysin (SYP; pre-synaptic protein) and the post-synaptic density protein 95 (PSD-95). Double immunostaining of GFP and SYP revealed no major differences in SYP immunoreactivity in untransduced, AAV9-GFP- or AAVrh10-GFP-transduced mouse hippocampi. In all cases, we observed a pattern of numerous small presynaptic vesicles in neurons (Fig. 4a–c). Similarly, anti-PSD-95 immunostaining revealing high abundance of PSD-95 positive vesicles also did not show major differences in the investigated conditions (Fig. 4d–f). Along these lines, western blot analysis of hippocampal biopsies also revealed no statistically significant differences in the levels of PSD-95 and SYP between non-injected mice or mice that received AAV9-GFP or AAVrh10-GFP (Fig. 4g–i).

### Co-injection of AAV9-GFP and AAVrh10-tdTomato leads to different transduction responses in adult mouse brain hippocampi

**Figure 5:**
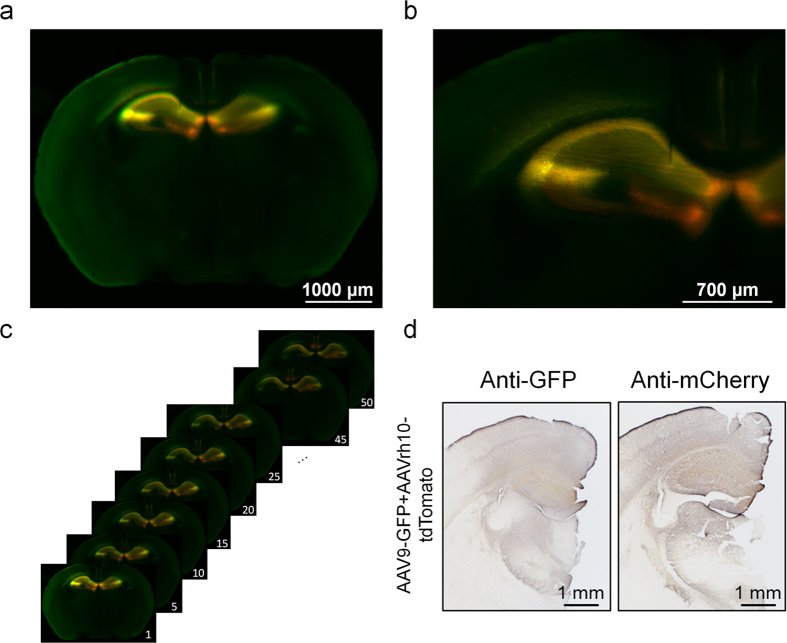
UM detects co-injected AAV9-GFP and AAVrh10-tdTomato in the adult mouse hippocampus. (**a**) AAV9-GFP and AAVrh10-tdTomato co-transduced whole adult mouse brains were cleared using the FluoClearBABB protocol and analysed with UM. Green and red fluorescence signals were clearly visible in CA1, CA2 and in the subiculum. An overlay between GFP- and tdTomato-fluorescence can be seen in the CA1 and CA2 cell layer. Fluorescence encoded by AAVrh10-tdTomato was more prominent in the subiculum. Fluorescence signals of the whole brain were detected with a 2-fold magnification. (**b**) 4-fold magnification of a. (**c**) Whole brain fluorescence analysis and video construction was obtained from rostral to caudal (1500 μm in 5 μm steps). The panel shows every 5^th^ out of a 50 steps from the measurement. The z-stack of a whole brain measurement can be used to create a video (Supplementary Video S1). (**d**) Representative immunohistochemically labelled coronal sections from C57BL/6J mice (n = 2) simultaneously co-injected with AAV9-GFP and AAVrh10-tdTomato (ratio 1/3) in the hippocampus. Brain slices were separately immunohistochemically labelled with an anti-GFP antibody or an anti-mCherry antibody (that also detects tdTomato) which are detected in the mouse hippocampus. Bars: 1 mm.

We next studied the effects of AAV9-GFP and AAVrh10-tdTomato co-injections in adult mouse brain hippocampi. To that aim, we performed measurements of the whole brains in order to detect both green (GFP) and red (tdTomato) fluorescence and generated overlay signals. Analysis revealed an overlap of GFP and tdTomato fluorescence that resulted in yellow signals in the CA1 and CA2 cell layers in the mouse hippocampus. Differences in transduction of brains were further detected in the subiculum. In this region, only red fluorescence was detected (Fig. 5a–c). Immunohistochemistry data support the results obtained with UM, showing stronger immunoreactivity with tdTomato as compared to GFP, overexpressed with AAVrh10 and AAV9, respectively (Fig. 5d). Whole brains were again scanned from cranial to caudal and single TIFF images were collected in order to create a video afterwards. The video allows traveling through fluorescence signal-transduced hippocampi of adult mouse brains. The resolution allows visualizing fluorescence-tagged cell compartments and distinct regions in the hippocampus. AAV9-GFP and AAVrh10-tdTomato transduce the same regions in the hippocampus except for the subiculum that was only transduced by AAVrh10-tdTomato (Fig. 5a,b). Taken together, these results suggest that UM is a highly sensitive technique that allows detecting differences in the transduction efficacies of different AAV serotypes in the mouse brain.

## Discussion

In this study we demonstrated that light sheet microscopy allows for a detailed preclinical mapping of viral transduction systems in undissected and optically cleared whole adult mouse brains with single cell resolution. With this platform we demonstrate that two alternative AAV serotypes (AAV9, AAVrh10) expressing fluorescent reporter proteins show almost similar expression patterns and efficiently transduce the mouse hippocampus, essentially neurons. UM revealed that AAV9 is slightly more efficient than AAVrh10 in mediating GFP reporter gene expression in neurons of the mouse hippocampus.

In the past, UM has been successfully applied on semi-transparent model organisms, i.e., zebrafish, Medaka and Drosophila^18^,^19^,^20^. With the advent of novel clearing procedures, imaging of whole organs and even whole brains is now possible^15^. Yet, these methods reveal differences in the maintenance of signals from proteinaceous reporter fluorophores such as GFP. To optically clear whole adult mouse brains, we have compared the well-established 3DISCO with the new FluoClearBABB clearing protocol^15^. FluoClearBABB enables clearing and maintenance of fluorescence signals even in undissected brains of aged mice. In contrast to 3DISCO-cleared brains, FluoClearBABB-cleared brains exhibit stronger reporter fluorescent levels and allowed for multiple measurements without loss of fluorescence signals, even after several months^15^. We re-scanned brains after storage at 4 °C and fluorescence signals were still intact after 2-3 months. In contrast, 3DISCO cleared brains show a decrease in fluorescence intensity already after 24 hours. Clearing with 3DISCO is less time-consuming than FluoClearBABB (five days in comparison to nine days), but the opportunity to re-image samples multiple times is a big advantage. Here, we used the UM platform to study transduction of CA pyramidal neurons and dentate granule neurons with AAV9 and AAVrh10 vectors. Data provided by UM suggests that AAV9 is a more efficient serotype than AAVrh10 in terms of mediating GFP reporter gene expression in mouse hippocampal neurons. These data highlight the sensitivity of UM, given that we did not find major differences in GFP expression mediated by AAV9 and AAVrh10 vectors in the mouse hippocampus, using standard western blot and immunofluorescence procedures. Recent studies applied laborious semi-quantitative methods, i.e., scoring systems to evaluate GFP immunoreactivity in different CNS regions^21^. However, these studies could not detect major differences in hippocampal transduction in the brain and spinal cord of neonatal mice when using AAV9 and AAVrh10 vectors^22^. Using UM, single transduced neurons in hippocampal regions CA1 to 4 were clearly visible which allows for detailed mapping of transduction efficacy.

Fifty years after the discovery of its parental virus, AAV vectors have evolved from an ordinary concomitant in culture to one of the most auspicious and frequently used vector systems for human gene therapy^23^. The combination of non-pathogenicity and efficient persistence of AAVs makes it an attractive delivery vehicle for gene therapy applications^24^. To date, twelve naturally occurring human AAV serotypes^25^ and more than one hundred serotypes from non-human primates and other species have been discovered^26^,^27^. The identification of novel serotypes, such as AAV9 and AAVrh10, directed under the action of various promoters, as well as the repertoire of engineered chimera vectors, are likely to improve and enlarge the AAV application range^22^,^28^,^29^. Further optimization of AAV vector design, which considers biological determinants such as AAV-cell- and region-specific tropism, is likely to overcome most of the obstacles for successful transduction, thus facilitating the widespread application of AAV for an even broader range of therapeutic applications^11^,^30^. With the steadily increasing number of viral gene therapy vectors and the growing number of potential clinical applications, an urgent need for fast, reliable and precise methods to assess viral tropism, transduction efficacy and persistence exists.

The combination of viral vector systems in transduced and transgenic reporter animal models allows for UM multiple colour-imaging to assess the specificity of cell-type or development-dependent reporter expression. We investigated reporter expression after co-injection of AAV9-GFP and AAVrh10-tdTomato in the adult mouse hippocampi and detected differences in the transduction of the subiculum, which was preferably transduced by AAVrh10-tdTomato. The CA1 and CA2 layer showed a similar transduction pattern with fluorescence signals and we saw a clear overlay of signals resulting in yellow fluorescence. In order to use AAVs as a shuttle for gene therapies, knowledge about the tropism of different viral vectors in the mammalian brain is important for treatment response and outcome. UM is a technique that allows for efficiently addressing these questions in unperturbed mammalian rodent brains on single cell level.

We agree that human brain-specific viral transduction patterns can only be modelled in an approximate manner when using rodents. However, we also believe that evaluating experimental therapies in animal models closer to the human system (i.e., non-human primates) will be instrumental for predicting therapeutic efficacy in human trials. UM is also suited to identify subanatomical transduction patterns in parts of these larger brains, which can be computationally assembled in the end to obtain a 3D reconstruction of whole brains. In summary, UM offers high-resolution 3D analysis of AAV-mediated fluorescent reporter gene expression in various cell types, such as neurons with intact anatomical projections deep inside the uncut adult rodent brain. Therefore, this method may open new avenues for the modelling of diseases in the mouse brain or to test therapeutic approaches in mouse models of neurodegenerative disorders.

## Methods

### Animals

Eight week-old conventional male C57BL/6J mice (Janvier, Le Genest St Isle, France) (n = 26) were used in this study. Animals were maintained in our animal facility under specific pathogen-free conditions. Mice were housed in a temperature-controlled room and maintained on a 12 h light/dark cycle. Food and water were available *ad libitum*. The experiments were carried out in accordance with the European Community Council directive (86/609/EEC) for the care and use of laboratory animals. All procedures were approved by the Regional Ethics Committee in Animal Experiment No. 44 of the Ile-de-France region.

### Stereotactic injections of AAVs

Mice were anesthetized by intraperitoneal injection of ketamine/xylazine (0.1/0.05 g/kg body weight) and positioned on a stereotactic frame (Stoelting, Wood Dale, USA). The green fluorescent protein (GFP) was driven by the hybrid cytomegalovirus enhancer/chicken beta-actin constitutive promoter (CAG) or the PGK-1 (phosphoglycerate kinase 1) promoter. The tdTomato protein is bright red fluorescent and also driven by the PGK-1 promoter. PGK-1 was used as a promoter for the fluorescent proteins in the two-colour experiments.

Both AAV9 and AAVrh10 vector stocks encoding the GFP or tdTomato protein were generated by transient transfection of 293T cells and purified by ultracentrifugation on a iodixanol discontinuous gradient^31^,^32^. The titre of both vectors was 7.5 × 10^12^ vector genomes per ml (vg/ml). Recombinant vectors (either AAV9-CAG-GFP, AAVrh10-CAG-GFP, AAV9-PGK-GFP or AAVrh10-PGK-tdTomato) were diluted in phosphate-buffered saline (PBS) 0.1 M and bilaterally injected into the hippocampus. Two microliters of viral preparation (corresponding to 2 × 10^9^ viral genomes (vg)) were injected into the left and right hippocampi at a rate of 0.2 μl/min. Two injections sites (1 μl+1 μl) per hippocampus were used to optimize virus spreading. Stereotactic coordinates of injection sites from *bregma* were: anteroposterior −2 mm; mediolateral +/−1 mm; dorsoventral −2.25 mm and anteroposterior −2 mm; mediolateral +/−1 mm; dorsoventral −1.75 mm. For co-injection of AAV9-PGK-GFP and AAVrh10-PGK-tdTomato, both AAV9-GFP (2.5 × 10^9^ vg) and AAVrh10-tdTomato (7.5 × 10^9^ vg) were mixed and co-injected in the mouse hippocampus using the procedure and stereotactic coordinates, described above.

### Tissue preparation

C57BL/6J mice were sacrificed six to eight weeks post-injection (age of animals: 3.5 to 4 months). The animals received an overdose of sodium pentobarbital and were transcardially perfused with 0.1 M PBS prior to brain extraction. For histological processing, the left cerebral hemisphere was dissected and post-fixed in 4% paraformaldehyde (PFA) in 0.1 M PBS for one week. Brains were cryoprotected by incubation in a 30% sucrose/0.1 M PBS solution. Coronal brain sections (40 μm) were cut on a freezing microtome (Leica, Wetzlar, Germany), collected serially, and stored at −20 °C until processing. The right hemisphere was dissected to extract the hippocampus for biochemical analysis. Samples were then homogenized in a lysis buffer (TBS, NaCl 150 mM and Triton 1%) containing phosphatase (Pierce, Rockford, IL, USA) and protease (Roche, Mannheim, Germany) inhibitors. After centrifugation (20 min, 13 000 rpm, 4 °C), the supernatant was collected and the protein concentration was quantified (BCA Protein Assay, Thermo Fisher Scientific, Waltham, USA). Lysate aliquots (3 mg of protein/ml) were stored at −80 °C until use.

### Western blot

Equal amounts of total protein extract (30 μg) were electrophoretically separated using SDS–PAGE in 4–12% Bis–Tris gels (NuPAGE^®^ Novex Bis-tris midi gel 15 or 26 wells, Life Technologies, Carlsbad, USA) and transferred to nitrocellulose membranes. Blocked membranes (5% non-fat dry milk in TBS/0.1% Tween-20) were incubated with the primary antibodies (Table 1) overnight at 4 °C, and washed three times with TBS/0.1% Tween-20 (T-BST) for 10 min. Membranes were then labelled with secondary anti-IgG-HRP antibodies raised against each corresponding primary antibody. After three washes with T-BST, the membranes were incubated with ECL chemiluminescent reagent (Clarity Western ECL substrate; GE Healthcare, Little Chalfont, UK) according to the instructions of the supplier. Peroxidase activity was detected with the camera system Fusion TX7 (Fisher Scientific). Normalization was done by densitometry analysis with the Quantity One 1D image analysis software (version 4.4; Biorad, Hercules, CA, USA). The optical densities were normalized with respect to a housekeeping protein (GAPDH, actin or tubulin). A partition ratio was calculated and normalized with respect to the sample with the highest value defined as one.

### Immunostaining

The immunohistochemical procedure was initiated by quenching endogenous peroxidase by incubating free-floating sections in hydrogen peroxide for 30 min at room temperature (RT). After three washes, slices were blocked in PBS/0.1% Triton X-100 containing 10% Normal Goat Serum (NGS, Gibco) for 1 h at RT. The sections were then incubated with the primary antibody (anti-GFP or anti-mCherry that also detects tdTomato) overnight at 4 °C. After three washings, the sections were incubated with the corresponding biotinylated secondary antibody (1:250; Vector Laboratories Inc., CA, USA) diluted in PBS/0.1% Triton X-100 and 10% NGS for 2 h, at RT. After three washes, bound antibodies were visualized by the ABC amplification system (Vectastain ABC kit, Vector Laboratories, West Grove, USA) and 3,3′-diaminobenzidine tetrahydrochloride (peroxidase substrate kit, DAB, Vector Laboratories, CA, USA) as the substrate. The sections were mounted, dehydrated by passing twice through ethanol and toluol solutions, and coverslipped with Eukitt (O. Kindler GmbH & CO, Freiburg, Germany). For immunofluorescence, slices were washed with 0.1 M PBS, permeabilized in PBS-Triton 0.1% before blocking in PBS/0.1% Triton X-100 containing 5% NGS for 1 h. Sections were then incubated with primary antibodies overnight at 4 °C. After three successive washes, brain slices were incubated for 2 h at RT with fluorescent secondary Alexa Fluor-conjugated antibodies (Invitrogen, Carlsbad, CA, USA). Slices were stained with DAPI (1:5000; Sigma, Darmstadt, Germany), mounted in Vectashield fluorescent mounting media (Vector laboratories, Burlingame, CA, USA) and conserved at 4 °C.

### Image acquisition

Images of immunostained sections were acquired with LAS V3.8 (Leica) software, at room temperature, with a bright field Leica DM 4000B microscope equipped with 10x/1.30 lenses and a Leica DFC500 digital camera. Confocal images were acquired with a Leica SP8 microscope equipped with 60x/1.35 lens. Photographs for comparison were taken under identical conditions of image acquisition and all adjustments of brightness and contrast were applied uniformly to all images.

### Brain slices from fixed brains for UM analysis

The mouse brains, fixed in 4% PFA in 0.1 M PBS were sectioned coronally into 250 μm thick slices using a vibratome (Leica VT 1000S) in cold PBS. Slices were collected in PBS in 6-well plates and then embedded in 2% agarose in a maximum volume of 600 μl. The agarose was cut in squares afterwards to fit in the sample holder for UM analysis.

### Clearing with 3DISCO and FluoClearBABB for UM analysis

For UM analysis, embedded slices and whole brains were optically cleared using organic solvents. Clearing was performed in accordance with the 3DISCO^17^ or with the FluoClearBABB protocol^15^.

For 3DISCO, brain slices were transferred into glass vials and the first clearing solution, 50% Tetrahydrofuran (THF; Sigma Aldrich), was gently added using a pipette. Vials were placed into black 50 ml Falcon tubes and then mounted onto an overhead turning wheel (program C3, 15 rpm, Neolab, intelli-mixer). Clearing was performed at RT. After 1 h, the 50% THF solution was exchanged by a 70% THF solution. Vials were again put into black Falcon tubes and mounted onto the turning wheel for another hour. The procedure was repeated with 80% and 100% THF solutions, respectively. Samples were incubated overnight in 100% THF, which was exchanged and added again for another hour and then 4 h in Dibenzyl ether (DBE; 98%, Sigma-Aldrich, Steinheim, Germany) as the last clearing solution. Whole brains were cleared using the same THF concentrations and the turning wheel. The incubation times per clearing solution were adjusted to 12 h. The last clearing step with DBE was adjusted from 4 to 48 h. To avoid degradation of the fluorescent signal, samples were imaged immediately after the clearing procedure.

The FluoClearBABB protocol is based on benzyl alcohol/benzyl benzoate clearing in combination with a basic pH, which is maintained throughout the clearing procedure. The protocol is especially suited for effective clearing of aged rodent brains. After dissection, brains were kept in PBS at 4 °C. For the dehydration of brains, analytical grade alcohol (t-butanol, Sigma-Aldrich, Steinheim, Germany) was diluted with double-distilled water. Brains were dehydrated using t-butanols ranging from 30% to 100%. The clearing solution BABB was prepared by mixing benzyl alcohol (Merck, Darmstadt, Germany; analytical grade) and benzyl benzoate (Sigma-Aldrich, Steinheim, Germany; “purissimum p.A.” grade) in a 1:2 volume ratio. The pH levels of dehydration and clearing solutions were adjusted using an InLab Science electrode suited for organic solvents (Mettler-Toledo). pH levels were adjusted with triethylamine (Sigma-Aldrich, Steinheim, Germany). Samples were kept in black 50 ml Falcon tubes and mounted onto a turning wheel for the complete clearing procedure. Afterwards, samples were stored at 4 °C and were imaged directly after clearing. Repetitive measurements were possible due to this fluorescence-conserving clearing method^15^,^33^.

### UM data acquisition

The cleared brain slices in agarose and whole brains were scanned with the UltraMicroscope II (LaVision BioTec GmbH, Bielefeld, Germany). As standard magnification, we used 1x and 2x with a 2x objective lens and a white light laser with a wavelength spectrum ranging from 400 to 2400 nm. For the detection of AAV9 and AAVrh10, we used a filter with excitation range 470/24 nm and emission range 525/50 nm. For tdTomato transfected AAVrh10, we used a filter with excitation range 545/25 nm and emission range 585/40 nm. We acquired z-stack measurements with 4 μm step size and a total range of 250 μm for a slice and 1500 to 2000 μm for the coronal measurements of the whole brain. We either imaged single photos from the whole brain or slices in tagged image file format (TIFF) or converted the z-stack in an Audio Video Interleave (AVI) video format. For AVIs of whole brain z-stacks, we used 351 pictures and 20 frames/second and a x-resolution of 720 pixels and y-resolution of 576 pixels.

For imaging of co-injections of AAV9-CAG-GFP and AAVrh10-CAG-Tomato, whole brains were used. For the detection of AAV9 and AAVrh10 transduction, we again used a filter with an excitation range of 470/24 nm and emission range of 525/50 nm, as well as an excitation range of 545/25 nm and emission range of 585/40 nm. For analysis of co-injected viruses encoding GFP and tdTomato, respectively, we normalized the photon counts (i.e., GFP = 1; tdTomato = 1/3) to compare the transduction efficacies of both viruses. We acquired z-stack measurements with 10 μm step size and a total range of 500 μm. Data were exported as TIFF images and overlays of green and red channel were created using FIJI^34^. Overlays were generated and exported as TIFF images or AVI video.

### Statistical analysis

Statistical analyses were defined with relation to the experimental design used. All data are presented as the mean ± SEM. Statistical analyses of optical densities (OD) on scanned western blot films and the percentage of co-localization in IF studies were analysed using Student’s t-test or one-way ANOVA followed by a *post-hoc* Fisher’s test. Significance thresholds were set at *p *< 0.05 for all tests. All analyses were performed using the software Statistica (StatSoft Inc., Tulsa, USA) or Prism (GraphPad Software, La Jolla, USA).

## Additional Information

**How to cite this article**: Alves, S. *et al.* Ultramicroscopy as a novel tool to unravel the tropism of AAV gene therapy vectors in the brain. *Sci. Rep.* **6**, 28272; doi: 10.1038/srep28272 (2016).

## Supplementary Material

Supplementary Video S1

Supplementary Information

## Figures and Tables

**Table 1:** Primary antibodies.

Primary antibodies	Source	WB	IHC
Rabbit anti-GFP	Abcam	1:5000	1:5000
Chicken anti-GFP	Abcam	–	1:5000
Rabbit anti-Glial Fibrillary Acidic Protein (GFAP)	Dako	1:5000	1:4000
Rabbit anti-Olig2	Millipore	–	1:2000
Rabbit anti-Ionized Calcium Binding Adapter Molecule 1 (IBA1)	Wako	–	1:3000
Mouse anti-NeuN	Millipore	–	1:1000
ALDH1L1	Abcam	1:1000	–
Rabbit-anti-TGF-β	Abcam	1:1000	–
Mouse-anti-HSP90	Enzo Life Technologies	1:2000	–
Rabbit anti-post synaptic density 95 (PSD-95)	Invitrogen	1:2000	1:1000
Mouse anti-synaptophysin	Santa Cruz	1:1000	1:500
Mouse anti-actin	Abcam	1:3000	–
Rabbit anti-tubulin	Abcam	1:5000	–
Mouse anti-mCherry	Clontech	1:2000	1:1000

Antibodies used in western blot and immunohistochemical analyses.
