# Supplementary material for: Ultramicroscopy as a novel tool to unravel the tropism of AAV gene therapy vectors in the brain
Source: Sci Rep. 2016 Jun 20;6:28272. doi: 10.1038/srep28272 (PMC4913310; doi:10.1038/srep28272)

Ultramicroscopy as a novel tool to unravel the tropism of AAV gene therapy vectors in the brain

Sandro Alves[1*], Julia Bode[2,3*], Alexis-Pierre Bemelmans[4,5], Christof von Kalle[6], Nathalie Cartier[*1♯] and Björn Tews[*2,3♯]

*equal contribution

[1]INSERM U1169/MIRCen CEA, Fontenay aux Roses 92265, France, Université Paris-Sud, Université Paris-Saclay, Orsay 91400, France.

[2]Schaller Research Group at the University of Heidelberg and the German Cancer Research Center (DKFZ), Im Neuenheimer Feld 581, 69120 Heidelberg, Germany.

[3]Molecular Mechanisms of Tumor Invasion (V077), DKFZ, Im Neuenheimer Feld 581, 69120 Heidelberg, Germany

[4]Commissariat à l´Energie Atomique et aux Energies Alternatives (CEA), Départment de la Recherche Fondamentale (DRF), Institut d´Imagerie Biomédicale (I2BM), Molecular Imaging Research Center (MIRCen), Fontenay-aux-Roses, France

[5]Centre National de la Recherche Scientifique (CNRS), Université Paris-Sud, Université Paris-Saclay, UMR 9199, Neurodegenerative Diseases Laboratory, Fontenay-aux Roses, France

[6]Department of Translational Oncology, National Center for Tumor Diseases (NCT) and German Cancer Research Center (DKFZ), Im Neuenheimer Feld 280, 69120 Heidelberg, Germany.

[♯] **Corresponding authors:**

Nathalie Cartier, MD

Hôpital Bicêtre

INSERM U986 / MIRCen

CEA Fontenay aux Roses, Bât 61

18 route du panorama – BP6

92265 Fontenay aux Roses, France

Email: Nathalie.cartier@inserm.fr

Björn Tews, PhD, MSc

Schaller Research Group at the University of Heidelberg and the DKFZ

Molecular Mechanisms of Tumor Invasion (V077)

Im Neuenheimer Feld 581

69120 Heidelberg, Germany

Email: B.Tews@dkfz-heidelberg.de

Supplementary Results, Figure legends and tables

AAV9 and AAVrh10 promoted GFP expression and does not trigger major glial responses in the mouse hippocampus

We next studied the effects of AAV-GFP expression on the activation of astrocytes and microglia. To this end, we performed double staining between GFP and GFAP in brain slices containing the hippocampus of adult mice transduced with AAV9-GFP or AAVrh10-GFP. The hippocampus of non-transduced mice was used as control. The analysis of GFAP immunofluorescence was similar among hippocampi of mice transduced with either AAV9-GFP or AAVrh10-GFP, which was also not significantly different from GFAP immunoreactivity present in the non-injected hippocampi (Supplementary Figure S2a-c). As expected, the western blot analysis of hippocampal biopsies from non-injected mice or mice injected with AAV9-GFP or AAVrh10-GFP also did not reveal statistically significant differences in the levels of GFAP expression (Supplementary Figure S2d and f). The astrocytic marker 10-formyltetrahydrofolate dehydrogenase (ALDH1L1) also showed no statistical significant differences in expression between all groups tested (Supplementary Fig. 2d and e). Similar data were observed in biopsies harvested from the cerebral cortex, where the levels of GFAP and ALDH1L1 remained unchanged (Supplementary Fig. S2g-i). Furthermore, the two microglial markers Iba1 and TGF-β also did not reveal statistical significant differences in the levels of these two proteins among hippocampi non-transduced or transduced with either AAV9-GFP or AAVrh10-GFP (Supplementary Fig. S2j-l). These data highlight the safety of AAV as a shuttle for expression of transgenes in the mouse brain.

Supplementary Video S1. UM can detect AAV9-GFP distribution in the adult mouse brain. Whole brain fluorescence analysis and video construction from rostral to caudal (1500 µm in 5 µm steps). Video was created using 10 frames/ sec.

Supplementary Figure S2. AAV9- and AAVrh10-mediated GFP overexpression does not induce major glial responses in the mouse hippocampus. (a-c) Representative laser confocal microscopy imaging double staining between overexpressed GFP and astrocytic GFP. No major differences were found between the hippocampus of non-injected mice and mice injected with AAV9-GFP or AAVrh10-GFP. Bars: 100 µm. Western blot of hippocampal lysates from non-injected mice or mice injected with AAV9-GFP or AAVrh10-GFP: No statistical significant differences were found in the levels of GFAP and ALDH1L1 (d-f). Student's T-test. Representative data from 5 mice/group. Western blot of lysates from the corresponding cerebral cortex showed no modifications in the levels of GFAP and ALDH1L1 (g-i). Student's t-test. Representative data from 5 mice/group. (g-i) Representative western blot of hippocampal lysates from non-injected mice or mice injected with AAV9-GFP or AAVrh10-GFP showing no statistical significant differences in the expression of the microglial markers Iba1 and TGF-β (j-l). Student's t-test. Representative data from 5 mice/group. Optical densitometries were normalized according to the amount of actin/tubulin loaded in the corresponding lane. A partition ratio was calculated and expressed as optical densitometry (arbitrary units) relative to the sample with highest value for the normalization control set at 1. Values are expressed as mean ± SEM.

Supplementary Figure S2

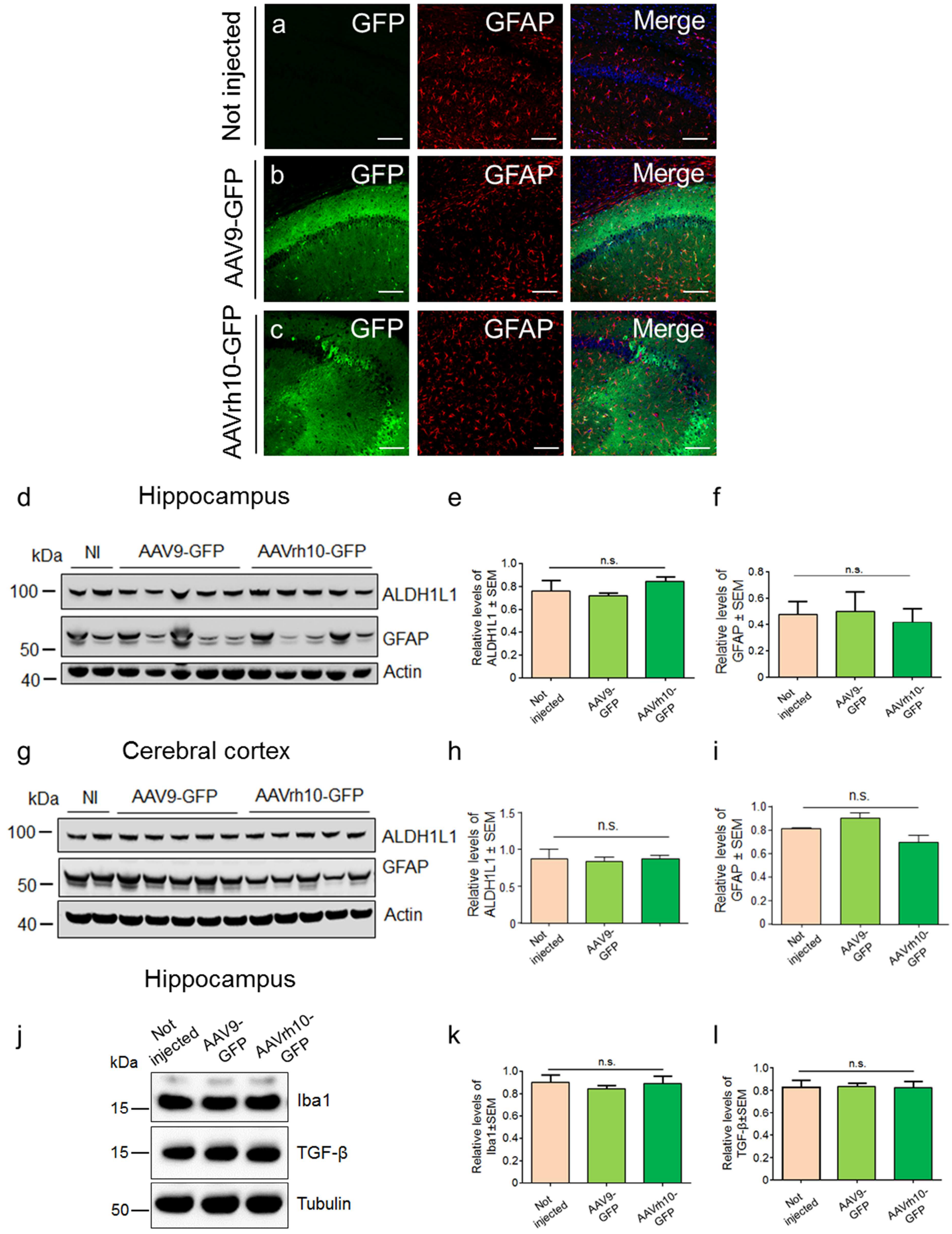

Supplement: Supplementary Information [file srep28272-s2.pdf]
